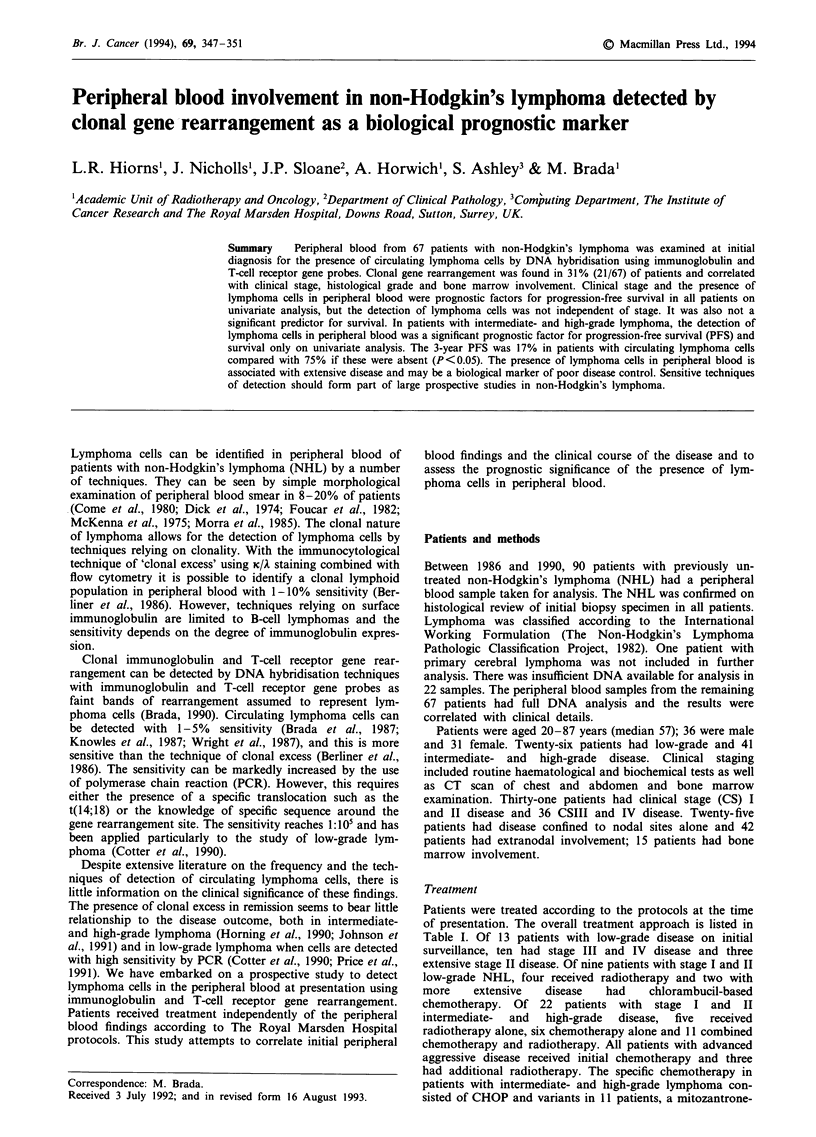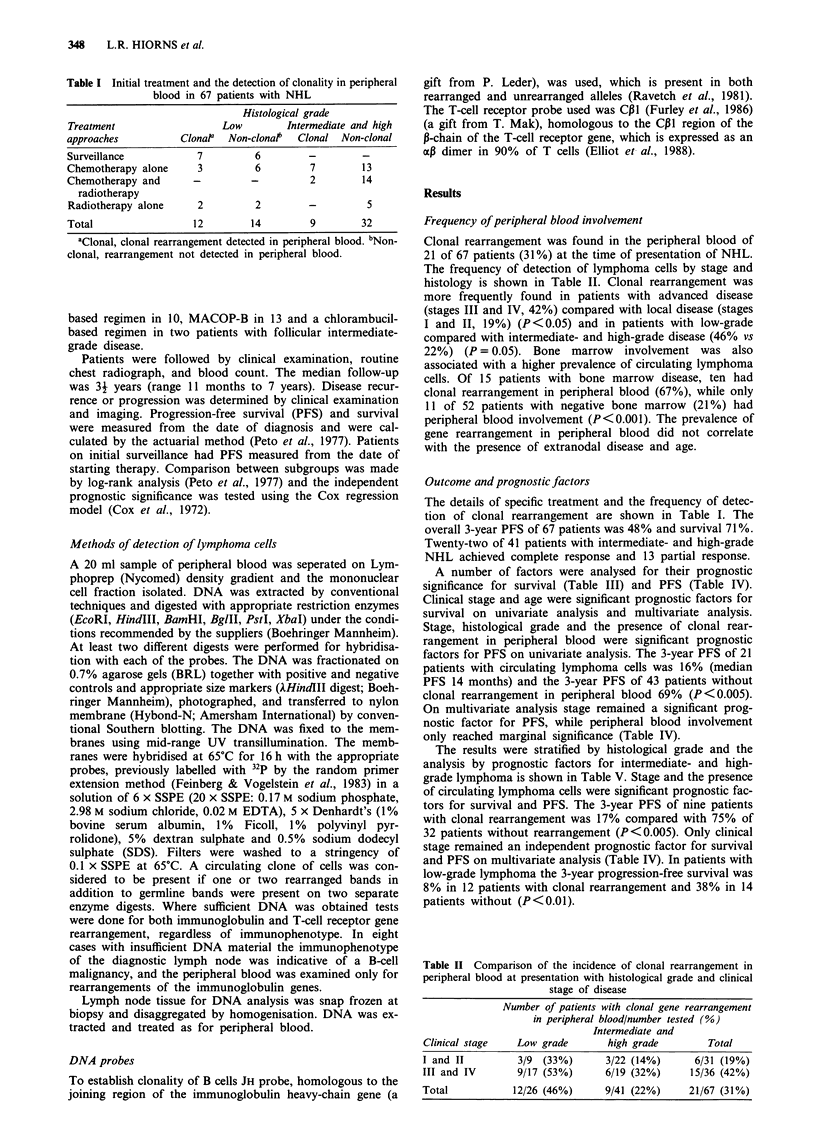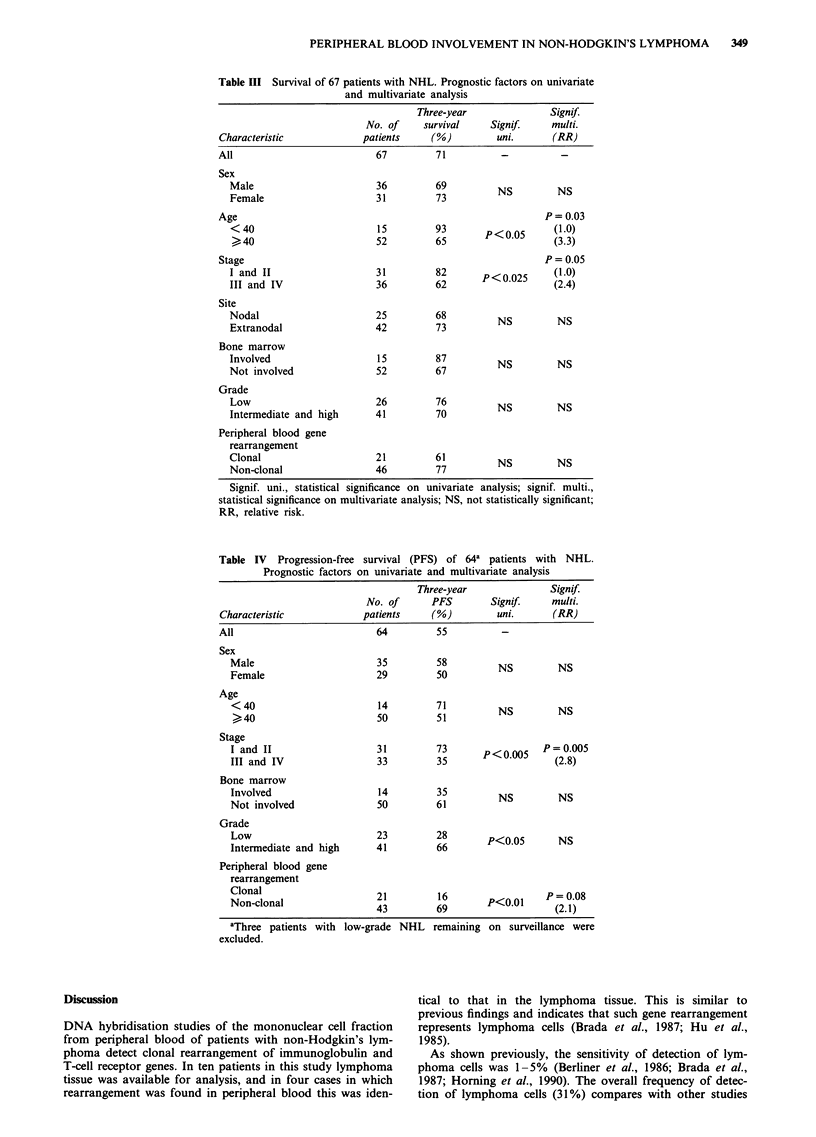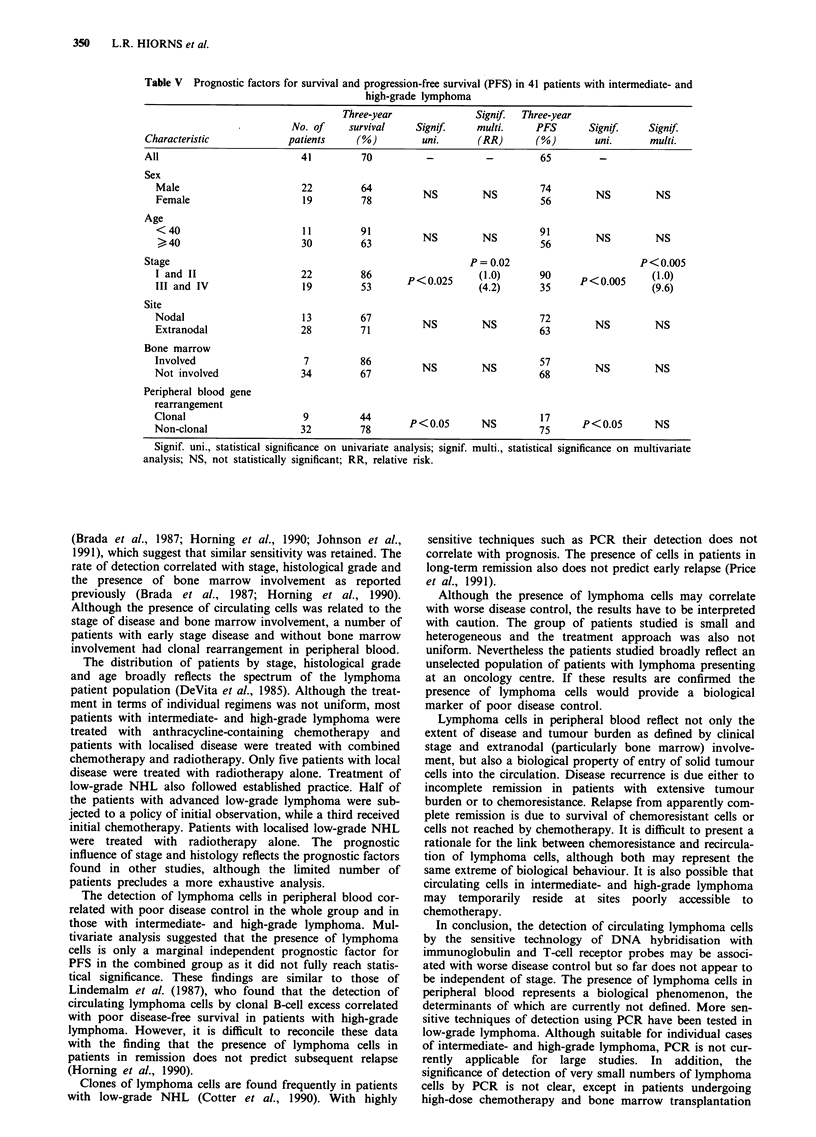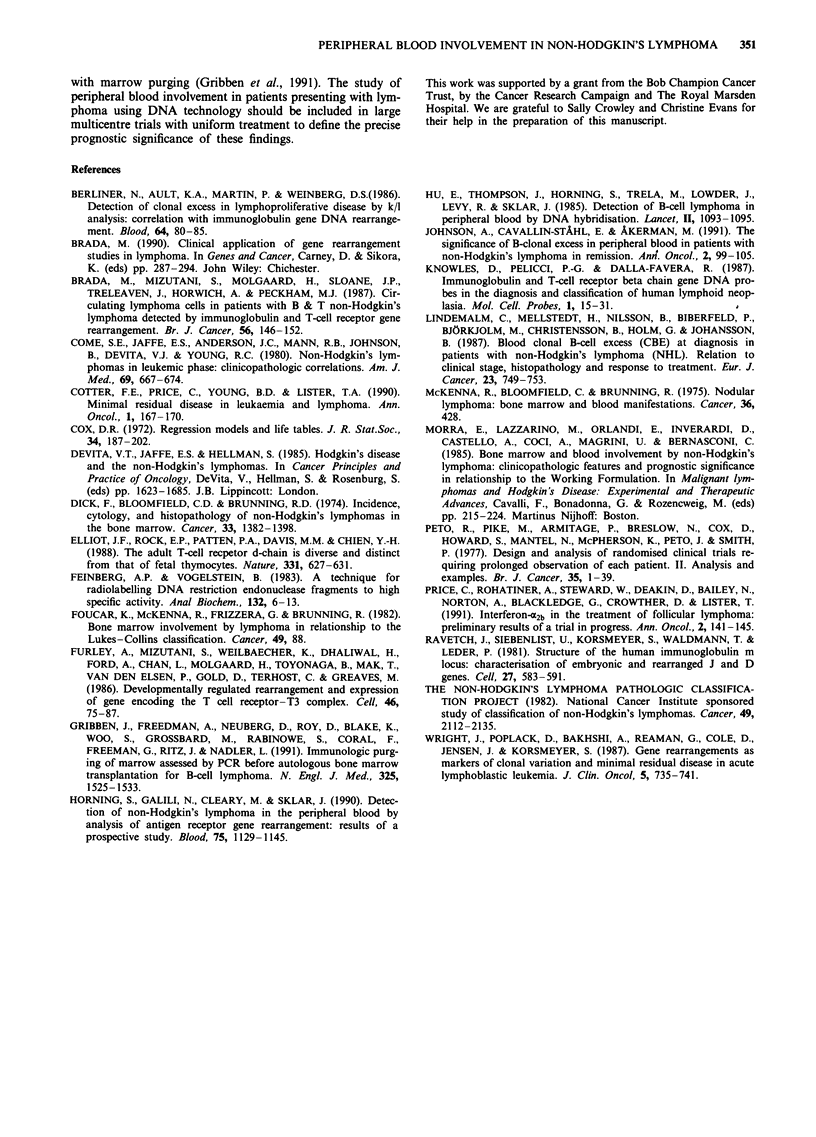# Peripheral blood involvement in non-Hodgkin's lymphoma detected by clonal gene rearrangement as a biological prognostic marker.

**DOI:** 10.1038/bjc.1994.63

**Published:** 1994-02

**Authors:** L. R. Hiorns, J. Nicholls, J. P. Sloane, A. Horwich, S. Ashley, M. Brada

**Affiliations:** Academic Unit of Radiotherapy and Oncology, Institute of Cancer Research, Sutton, Surrey, UK.

## Abstract

Peripheral blood from 67 patients with non-Hodgkin's lymphoma was examined at initial diagnosis for the presence of circulating lymphoma cells by DNA hybridisation using immunoglobulin and T-cell receptor gene probes. Clonal gene rearrangement was found in 31% (21/67) of patients and correlated with clinical stage, histological grade and bone marrow involvement. Clinical stage and the presence of lymphoma cells in peripheral blood were prognostic factors for progression-free survival in all patients on univariate analysis, but the detection of lymphoma cells was not independent of stage. It was also not a significant predictor for survival. In patients with intermediate- and high-grade lymphoma, the detection of lymphoma cells in peripheral blood was a significant prognostic factor for progression-free survival (PFS) and survival only on univariate analysis. The 3-year PFS was 17% in patients with circulating lymphoma cells compared with 75% if these were absent (P < 0.05). The presence of lymphoma cells in peripheral blood is associated with extensive disease and may be a biological marker of poor disease control. Sensitive techniques of detection should form part of large prospective studies in non-Hodgkin's lymphoma.


*Br. J. Cancer* (1994), **69**, 347–351 © Macmillan Press Ltd., 1994

### Peripheral blood involvement in non-Hodgkin's lymphoma detected by clonal gene rearrangement as a biological prognostic marker

L.R. Hiorns[1], J. Nicholls[1], J.P. Sloane[2], A. Horwich[1], S. Ashley[3] & M. Brada[1]

[1]*Academic Unit of Radiotherapy and Oncology,* [2]*Department of Clinical Pathology,* [3]*Computing Department, The Institute of Cancer Research and The Royal Marsden Hospital, Downs Road, Sutton, Surrey, UK.*

**Summary** Peripheral blood from 67 patients with non-Hodgkin's lymphoma was examined at initial diagnosis for the presence of circulating lymphoma cells by DNA hybridisation using immunoglobulin and T-cell receptor gene probes. Clonal gene rearrangement was found in 31% (21/67) of patients and correlated with clinical stage, histological grade and bone marrow involvement. Clinical stage and the presence of lymphoma cells in peripheral blood were prognostic factors for progression-free survival in all patients on univariate analysis, but the detection of lymphoma cells was not independent of stage. It was also not a significant predictor for survival. In patients with intermediate- and high-grade lymphoma, the detection of lymphoma cells in peripheral blood was a significant prognostic factor for progression-free survival (PFS) and survival only on univariate analysis. The 3-year PFS was 17% in patients with circulating lymphoma cells compared with 75% if these were absent ($P < 0.05$). The presence of lymphoma cells in peripheral blood is associated with extensive disease and may be a biological marker of poor disease control. Sensitive techniques of detection should form part of large prospective studies in non-Hodgkin's lymphoma.

Lymphoma cells can be identified in peripheral blood of patients with non-Hodgkin's lymphoma (NHL) by a number of techniques. They can be seen by simple morphological examination of peripheral blood smear in 8–20% of patients (Come *et al.*, 1980; Dick *et al.*, 1974; Foucar *et al.*, 1982; McKenna *et al.*, 1975; Morra *et al.*, 1985). The clonal nature of lymphoma allows for the detection of lymphoma cells by techniques relying on clonality. With the immunocytological technique of 'clonal excess' using $\kappa/\lambda$ staining combined with flow cytometry it is possible to identify a clonal lymphoid population in peripheral blood with 1–10% sensitivity (Berliner *et al.*, 1986). However, techniques relying on surface immunoglobulin are limited to B-cell lymphomas and the sensitivity depends on the degree of immunoglobulin expression.

Clonal immunoglobulin and T-cell receptor gene rearrangement can be detected by DNA hybridisation techniques with immunoglobulin and T-cell receptor gene probes as faint bands of rearrangement assumed to represent lymphoma cells (Brada, 1990). Circulating lymphoma cells can be detected with 1–5% sensitivity (Brada *et al.*, 1987; Knowles *et al.*, 1987; Wright *et al.*, 1987), and this is more sensitive than the technique of clonal excess (Berliner *et al.*, 1986). The sensitivity can be markedly increased by the use of polymerase chain reaction (PCR). However, this requires either the presence of a specific translocation such as the t(14;18) or the knowledge of specific sequence around the gene rearrangement site. The sensitivity reaches $1{:}10^5$ and has been applied particularly to the study of low-grade lymphoma (Cotter *et al.*, 1990).

Despite extensive literature on the frequency and the techniques of detection of circulating lymphoma cells, there is little information on the clinical significance of these findings. The presence of clonal excess in remission seems to bear little relationship to the disease outcome, both in intermediate- and high-grade lymphoma (Horning *et al.*, 1990; Johnson *et al.*, 1991) and in low-grade lymphoma when cells are detected with high sensitivity by PCR (Cotter *et al.*, 1990; Price *et al.*, 1991). We have embarked on a prospective study to detect lymphoma cells in the peripheral blood at presentation using immunoglobulin and T-cell receptor gene rearrangement. Patients received treatment independently of the peripheral blood findings according to The Royal Marsden Hospital protocols. This study attempts to correlate initial peripheral blood findings and the clinical course of the disease and to assess the prognostic significance of the presence of lymphoma cells in peripheral blood.

### Patients and methods

Between 1986 and 1990, 90 patients with previously untreated non-Hodgkin's lymphoma (NHL) had a peripheral blood sample taken for analysis. The NHL was confirmed on histological review of initial biopsy specimen in all patients. Lymphoma was classified according to the International Working Formulation (The Non-Hodgkin's Lymphoma Pathologic Classification Project, 1982). One patient with primary cerebral lymphoma was not included in further analysis. There was insufficient DNA available for analysis in 22 samples. The peripheral blood samples from the remaining 67 patients had full DNA analysis and the results were correlated with clinical details.

Patients were aged 20–87 years (median 57); 36 were male and 31 female. Twenty-six patients had low-grade and 41 intermediate- and high-grade disease. Clinical staging included routine haematological and biochemical tests as well as CT scan of chest and abdomen and bone marrow examination. Thirty-one patients had clinical stage (CS) I and II disease and 36 CSIII and IV disease. Twenty-five patients had disease confined to nodal sites alone and 42 patients had extranodal involvement; 15 patients had bone marrow involvement.

### *Treatment*

Patients were treated according to the protocols at the time of presentation. The overall treatment approach is listed in Table I. Of 13 patients with low-grade disease on initial surveillance, ten had stage III and IV disease and three extensive stage II disease. Of nine patients with stage I and II low-grade NHL, four received radiotherapy and two with more extensive disease had chlorambucil-based chemotherapy. Of 22 patients with stage I and II intermediate- and high-grade disease, five received radiotherapy alone, six chemotherapy alone and 11 combined chemotherapy and radiotherapy. All patients with advanced aggressive disease received initial chemotherapy and three had additional radiotherapy. The specific chemotherapy in patients with intermediate- and high-grade lymphoma consisted of CHOP and variants in 11 patients, a mitozantrone-

Correspondence: M. Brada.
Received 3 July 1992; and in revised form 16 August 1993.

**Table I** Initial treatment and the detection of clonality in peripheral blood in 67 patients with NHL

| *Treatment approaches* | *Histological grade* Low Clonal[a] | Low Non-clonal[b] | Intermediate and high Clonal | Intermediate and high Non-clonal |
|---|---|---|---|---|
| Surveillance | 7 | 6 | – | – |
| Chemotherapy alone | 3 | 6 | 7 | 13 |
| Chemotherapy and radiotherapy | – | – | 2 | 14 |
| Radiotherapy alone | 2 | 2 | – | 5 |
| Total | 12 | 14 | 9 | 32 |

[a]Clonal, clonal rearrangement detected in peripheral blood. [b]Non-clonal, rearrangement not detected in peripheral blood.

based regimen in 10, MACOP-B in 13 and a chlorambucil-based regimen in two patients with follicular intermediate-grade disease.

Patients were followed by clinical examination, routine chest radiograph, and blood count. The median follow-up was 3½ years (range 11 months to 7 years). Disease recurrence or progression was determined by clinical examination and imaging. Progression-free survival (PFS) and survival were measured from the date of diagnosis and were calculated by the actuarial method (Peto *et al.*, 1977). Patients on initial surveillance had PFS measured from the date of starting therapy. Comparison between subgroups was made by log-rank analysis (Peto *et al.*, 1977) and the independent prognostic significance was tested using the Cox regression model (Cox *et al.*, 1972).

*Methods of detection of lymphoma cells*

A 20 ml sample of peripheral blood was seperated on Lymphoprep (Nycomed) density gradient and the mononuclear cell fraction isolated. DNA was extracted by conventional techniques and digested with appropriate restriction enzymes (*Eco*RI, *Hind*III, *Bam*HI, *Bgl*II, *Pst*I, *Xba*I) under the conditions recommended by the suppliers (Boehringer Mannheim). At least two different digests were performed for hybridisation with each of the probes. The DNA was fractionated on 0.7% agarose gels (BRL) together with positive and negative controls and appropriate size markers ($\lambda$*Hind*III digest; Boehringer Mannheim), photographed, and transferred to nylon membrane (Hybond-N; Amersham International) by conventional Southern blotting. The DNA was fixed to the membranes using mid-range UV transillumination. The membranes were hybridised at 65°C for 16 h with the appropriate probes, previously labelled with $^{32}P$ by the random primer extension method (Feinberg & Vogelstein *et al.*, 1983) in a solution of 6 × SSPE (20 × SSPE: 0.17 M sodium phosphate, 2.98 M sodium chloride, 0.02 M EDTA), 5 × Denhardt's (1% bovine serum albumin, 1% Ficoll, 1% polyvinyl pyrrolidone), 5% dextran sulphate and 0.5% sodium dodecyl sulphate (SDS). Filters were washed to a stringency of 0.1 × SSPE at 65°C. A circulating clone of cells was considered to be present if one or two rearranged bands in addition to germline bands were present on two separate enzyme digests. Where sufficient DNA was obtained tests were done for both immunoglobulin and T-cell receptor gene rearrangement, regardless of immunophenotype. In eight cases with insufficient DNA material the immunophenotype of the diagnostic lymph node was indicative of a B-cell malignancy, and the peripheral blood was examined only for rearrangements of the immunoglobulin genes.

Lymph node tissue for DNA analysis was snap frozen at biopsy and disaggregated by homogenisation. DNA was extracted and treated as for peripheral blood.

*DNA probes*

To establish clonality of B cells JH probe, homologous to the joining region of the immunoglobulin heavy-chain gene (a gift from P. Leder), was used, which is present in both rearranged and unrearranged alleles (Ravetch *et al.*, 1981). The T-cell receptor probe used was C$\beta$1 (Furley *et al.*, 1986) (a gift from T. Mak), homologous to the C$\beta$1 region of the $\beta$-chain of the T-cell receptor gene, which is expressed as an $\alpha\beta$ dimer in 90% of T cells (Elliot *et al.*, 1988).

### Results

*Frequency of peripheral blood involvement*

Clonal rearrangement was found in the peripheral blood of 21 of 67 patients (31%) at the time of presentation of NHL. The frequency of detection of lymphoma cells by stage and histology is shown in Table II. Clonal rearrangement was more frequently found in patients with advanced disease (stages III and IV, 42%) compared with local disease (stages I and II, 19%) ($P<0.05$) and in patients with low-grade compared with intermediate- and high-grade disease (46% *vs* 22%) ($P=0.05$). Bone marrow involvement was also associated with a higher prevalence of circulating lymphoma cells. Of 15 patients with bone marrow disease, ten had clonal rearrangement in peripheral blood (67%), while only 11 of 52 patients with negative bone marrow (21%) had peripheral blood involvement ($P<0.001$). The prevalence of gene rearrangement in peripheral blood did not correlate with the presence of extranodal disease and age.

*Outcome and prognostic factors*

The details of specific treatment and the frequency of detection of clonal rearrangement are shown in Table I. The overall 3-year PFS of 67 patients was 48% and survival 71%. Twenty-two of 41 patients with intermediate- and high-grade NHL achieved complete response and 13 partial response.

A number of factors were analysed for their prognostic significance for survival (Table III) and PFS (Table IV). Clinical stage and age were significant prognostic factors for survival on univariate analysis and multivariate analysis. Stage, histological grade and the presence of clonal rearrangement in peripheral blood were significant prognostic factors for PFS on univariate analysis. The 3-year PFS of 21 patients with circulating lymphoma cells was 16% (median PFS 14 months) and the 3-year PFS of 43 patients without clonal rearrangement in peripheral blood 69% ($P<0.005$). On multivariate analysis stage remained a significant prognostic factor for PFS, while peripheral blood involvement only reached marginal significance (Table IV).

The results were stratified by histological grade and the analysis by prognostic factors for intermediate- and high-grade lymphoma is shown in Table V. Stage and the presence of circulating lymphoma cells were significant prognostic factors for survival and PFS. The 3-year PFS of nine patients with clonal rearrangement was 17% compared with 75% of 32 patients without rearrangement ($P<0.005$). Only clinical stage remained an independent prognostic factor for survival and PFS on multivariate analysis (Table IV). In patients with low-grade lymphoma the 3-year progression-free survival was 8% in 12 patients with clonal rearrangement and 38% in 14 patients without ($P<0.01$).

**Table II** Comparison of the incidence of clonal rearrangement in peripheral blood at presentation with histological grade and clinical stage of disease

| *Clinical stage* | *Number of patients with clonal gene rearrangement in peripheral blood/number tested (%)* Low grade | Intermediate and high grade | Total |
|---|---|---|---|
| I and II | 3/9 (33%) | 3/22 (14%) | 6/31 (19%) |
| III and IV | 9/17 (53%) | 6/19 (32%) | 15/36 (42%) |
| Total | 12/26 (46%) | 9/41 (22%) | 21/67 (31%) |

**Table III** Survival of 67 patients with NHL. Prognostic factors on univariate and multivariate analysis

| *Characteristic* | *No. of patients* | *Three-year survival (%)* | *Signif. uni.* | *Signif. multi. (RR)* |
|---|---|---|---|---|
| All | 67 | 71 | – | – |
| Sex | | | | |
| Male | 36 | 69 | NS | NS |
| Female | 31 | 73 | | |
| Age | | | | $P = 0.03$ |
| <40 | 15 | 93 | $P<0.05$ | (1.0) |
| ⩾40 | 52 | 65 | | (3.3) |
| Stage | | | | $P = 0.05$ |
| I and II | 31 | 82 | $P<0.025$ | (1.0) |
| III and IV | 36 | 62 | | (2.4) |
| Site | | | | |
| Nodal | 25 | 68 | NS | NS |
| Extranodal | 42 | 73 | | |
| Bone marrow | | | | |
| Involved | 15 | 87 | NS | NS |
| Not involved | 52 | 67 | | |
| Grade | | | | |
| Low | 26 | 76 | NS | NS |
| Intermediate and high | 41 | 70 | | |
| Peripheral blood gene rearrangement | | | | |
| Clonal | 21 | 61 | NS | NS |
| Non-clonal | 46 | 77 | | |

Signif. uni., statistical significance on univariate analysis; signif. multi., statistical significance on multivariate analysis; NS, not statistically significant; RR, relative risk.

**Table IV** Progression-free survival (PFS) of 64[a] patients with NHL. Prognostic factors on univariate and multivariate analysis

| *Characteristic* | *No. of patients* | *Three-year PFS (%)* | *Signif. uni.* | *Signif. multi. (RR)* |
|---|---|---|---|---|
| All | 64 | 55 | – | |
| Sex | | | | |
| Male | 35 | 58 | NS | NS |
| Female | 29 | 50 | | |
| Age | | | | |
| <40 | 14 | 71 | NS | NS |
| ⩾40 | 50 | 51 | | |
| Stage | | | | |
| I and II | 31 | 73 | $P<0.005$ | $P = 0.005$ |
| III and IV | 33 | 35 | | (2.8) |
| Bone marrow | | | | |
| Involved | 14 | 35 | NS | NS |
| Not involved | 50 | 61 | | |
| Grade | | | | |
| Low | 23 | 28 | $P<0.05$ | NS |
| Intermediate and high | 41 | 66 | | |
| Peripheral blood gene rearrangement | | | | |
| Clonal | 21 | 16 | $P<0.01$ | $P = 0.08$ |
| Non-clonal | 43 | 69 | | (2.1) |

[a]Three patients with low-grade NHL remaining on surveillance were excluded.

### Discussion

DNA hybridisation studies of the mononuclear cell fraction from peripheral blood of patients with non-Hodgkin's lymphoma detect clonal rearrangement of immunoglobulin and T-cell receptor genes. In ten patients in this study lymphoma tissue was available for analysis, and in four cases in which rearrangement was found in peripheral blood this was identical to that in the lymphoma tissue. This is similar to previous findings and indicates that such gene rearrangement represents lymphoma cells (Brada *et al.*, 1987; Hu *et al.*, 1985).

As shown previously, the sensitivity of detection of lymphoma cells was 1–5% (Berliner *et al.*, 1986; Brada *et al.*, 1987; Horning *et al.*, 1990). The overall frequency of detection of lymphoma cells (31%) compares with other studies

**Table V** Prognostic factors for survival and progression-free survival (PFS) in 41 patients with intermediate- and high-grade lymphoma

| *Characteristic* | *No. of patients* | *Three-year survival (%)* | *Signif. uni.* | *Signif. multi. (RR)* | *Three-year PFS (%)* | *Signif. uni.* | *Signif. multi.* |
|---|---|---|---|---|---|---|---|
| All | 41 | 70 | – | – | 65 | – | |
| Sex | | | | | | | |
| Male | 22 | 64 | NS | NS | 74 | NS | NS |
| Female | 19 | 78 | | | 56 | | |
| Age | | | | | | | |
| $<40$ | 11 | 91 | NS | NS | 91 | NS | NS |
| $\geqslant 40$ | 30 | 63 | | | 56 | | |
| Stage | | | | $P = 0.02$ | | | $P < 0.005$ |
| I and II | 22 | 86 | $P < 0.025$ | (1.0) | 90 | $P < 0.005$ | (1.0) |
| III and IV | 19 | 53 | | (4.2) | 35 | | (9.6) |
| Site | | | | | | | |
| Nodal | 13 | 67 | NS | NS | 72 | NS | NS |
| Extranodal | 28 | 71 | | | 63 | | |
| Bone marrow | | | | | | | |
| Involved | 7 | 86 | NS | NS | 57 | NS | NS |
| Not involved | 34 | 67 | | | 68 | | |
| Peripheral blood gene rearrangement | | | | | | | |
| Clonal | 9 | 44 | $P < 0.05$ | NS | 17 | $P < 0.05$ | NS |
| Non-clonal | 32 | 78 | | | 75 | | |

Signif. uni., statistical significance on univariate analysis; signif. multi., statistical significance on multivariate analysis; NS, not statistically significant; RR, relative risk.

(Brada *et al.*, 1987; Horning *et al.*, 1990; Johnson *et al.*, 1991), which suggest that similar sensitivity was retained. The rate of detection correlated with stage, histological grade and the presence of bone marrow involvement as reported previously (Brada *et al.*, 1987; Horning *et al.*, 1990). Although the presence of circulating cells was related to the stage of disease and bone marrow involvement, a number of patients with early stage disease and without bone marrow involvement had clonal rearrangement in peripheral blood.

The distribution of patients by stage, histological grade and age broadly reflects the spectrum of the lymphoma patient population (DeVita *et al.*, 1985). Although the treatment in terms of individual regimens was not uniform, most patients with intermediate- and high-grade lymphoma were treated with anthracycline-containing chemotherapy and patients with localised disease were treated with combined chemotherapy and radiotherapy. Only five patients with local disease were treated with radiotherapy alone. Treatment of low-grade NHL also followed established practice. Half of the patients with advanced low-grade lymphoma were subjected to a policy of initial observation, while a third received initial chemotherapy. Patients with localised low-grade NHL were treated with radiotherapy alone. The prognostic influence of stage and histology reflects the prognostic factors found in other studies, although the limited number of patients precludes a more exhaustive analysis.

The detection of lymphoma cells in peripheral blood correlated with poor disease control in the whole group and in those with intermediate- and high-grade lymphoma. Multivariate analysis suggested that the presence of lymphoma cells is only a marginal independent prognostic factor for PFS in the combined group as it did not fully reach statistical significance. These findings are similar to those of Lindemalm *et al.* (1987), who found that the detection of circulating lymphoma cells by clonal B-cell excess correlated with poor disease-free survival in patients with high-grade lymphoma. However, it is difficult to reconcile these data with the finding that the presence of lymphoma cells in patients in remission does not predict subsequent relapse (Horning *et al.*, 1990).

Clones of lymphoma cells are found frequently in patients with low-grade NHL (Cotter *et al.*, 1990). With highly sensitive techniques such as PCR their detection does not correlate with prognosis. The presence of cells in patients in long-term remission also does not predict early relapse (Price *et al.*, 1991).

Although the presence of lymphoma cells may correlate with worse disease control, the results have to be interpreted with caution. The group of patients studied is small and heterogeneous and the treatment approach was also not uniform. Nevertheless the patients studied broadly reflect an unselected population of patients with lymphoma presenting at an oncology centre. If these results are confirmed the presence of lymphoma cells would provide a biological marker of poor disease control.

Lymphoma cells in peripheral blood reflect not only the extent of disease and tumour burden as defined by clinical stage and extranodal (particularly bone marrow) involvement, but also a biological property of entry of solid tumour cells into the circulation. Disease recurrence is due either to incomplete remission in patients with extensive tumour burden or to chemoresistance. Relapse from apparently complete remission is due to survival of chemoresistant cells or cells not reached by chemotherapy. It is difficult to present a rationale for the link between chemoresistance and recirculation of lymphoma cells, although both may represent the same extreme of biological behaviour. It is also possible that circulating cells in intermediate- and high-grade lymphoma may temporarily reside at sites poorly accessible to chemotherapy.

In conclusion, the detection of circulating lymphoma cells by the sensitive technology of DNA hybridisation with immunoglobulin and T-cell receptor probes may be associated with worse disease control but so far does not appear to be independent of stage. The presence of lymphoma cells in peripheral blood represents a biological phenomenon, the determinants of which are currently not defined. More sensitive techniques of detection using PCR have been tested in low-grade lymphoma. Although suitable for individual cases of intermediate- and high-grade lymphoma, PCR is not currently applicable for large studies. In addition, the significance of detection of very small numbers of lymphoma cells by PCR is not clear, except in patients undergoing high-dose chemotherapy and bone marrow transplantation with marrow purging (Gribben *et al.*, 1991). The study of peripheral blood involvement in patients presenting with lymphoma using DNA technology should be included in large multicentre trials with uniform treatment to define the precise prognostic significance of these findings.

This work was supported by a grant from the Bob Champion Cancer Trust, by the Cancer Research Campaign and The Royal Marsden Hospital. We are grateful to Sally Crowley and Christine Evans for their help in the preparation of this manuscript.

### References

BERLINER, N., AULT, K.A., MARTIN, P. & WEINBERG, D.S.(1986). Detection of clonal excess in lymphoproliferative disease by k/l analysis: correlation with immunoglobulin gene DNA rearrangement. *Blood*, **64**, 80-85.

BRADA, M. (1990). Clinical application of gene rearrangement studies in lymphoma. In *Genes and Cancer*, Carney, D. & Sikora, K. (eds) pp. 287-294. John Wiley: Chichester.

BRADA, M., MIZUTANI, S., MOLGAARD, H., SLOANE, J.P., TRELEAVEN, J., HORWICH, A. & PECKHAM, M.J. (1987). Circulating lymphoma cells in patients with B & T non-Hodgkin's lymphoma detected by immunoglobulin and T-cell receptor gene rearrangement. *Br. J. Cancer*, **56**, 146-152.

COME, S.E., JAFFE, E.S., ANDERSON, J.C., MANN, R.B., JOHNSON, B., DEVITA, V.J. & YOUNG, R.C. (1980). Non-Hodgkin's lymphomas in leukemic phase: clinicopathologic correlations. *Am. J. Med.*, **69**, 667-674.

COTTER, F.E., PRICE, C., YOUNG, B.D. & LISTER, T.A. (1990). Minimal residual disease in leukaemia and lymphoma. *Ann. Oncol.*, **1**, 167-170.

COX, D.R. (1972). Regression models and life tables. *J. R. Stat.Soc.*, **34**, 187-202.

DEVITA, V.T., JAFFE, E.S. & HELLMAN, S. (1985). Hodgkin's disease and the non-Hodgkin's lymphomas. In *Cancer Principles and Practice of Oncology*, DeVita, V., Hellman, S. & Rosenburg, S. (eds) pp. 1623-1685. J.B. Lippincott: London.

DICK, F., BLOOMFIELD, C.D. & BRUNNING, R.D. (1974). Incidence, cytology, and histopathology of non-Hodgkin's lymphomas in the bone marrow. *Cancer*, **33**, 1382-1398.

ELLIOT, J.F., ROCK, E.P., PATTEN, P.A., DAVIS, M.M. & CHIEN, Y.-H. (1988). The adult T-cell recpetor d-chain is diverse and distinct from that of fetal thymocytes. *Nature*, **331**, 627-631.

FEINBERG, A.P. & VOGELSTEIN, B. (1983). A technique for radiolabelling DNA restriction endonuclease fragments to high specific activity. *Anal Biochem.*, **132**, 6-13.

FOUCAR, K., McKENNA, R., FRIZZERA, G. & BRUNNING, R. (1982). Bone marrow involvement by lymphoma in relationship to the Lukes-Collins classification. *Cancer*, **49**, 88.

FURLEY, A., MIZUTANI, S., WEILBAECHER, K., DHALIWAL, H., FORD, A., CHAN, L., MOLGAARD, H., TOYONAGA, B., MAK, T., VAN DEN ELSEN, P., GOLD, D., TERHOST, C. & GREAVES, M. (1986). Developmentally regulated rearrangement and expression of gene encoding the T cell receptor-T3 complex. *Cell*, **46**, 75-87.

GRIBBEN, J., FREEDMAN, A., NEUBERG, D., ROY, D., BLAKE, K., WOO, S., GROSSBARD, M., RABINOWE, S., CORAL, F., FREEMAN, G., RITZ, J. & NADLER, L. (1991). Immunologic purging of marrow assessed by PCR before autologous bone marrow transplantation for B-cell lymphoma. *N. Engl. J. Med.*, **325**, 1525-1533.

HORNING, S., GALILI, N., CLEARY, M. & SKLAR, J. (1990). Detection of non-Hodgkin's lymphoma in the peripheral blood by analysis of antigen receptor gene rearrangement: results of a prospective study. *Blood*, **75**, 1129-1145.

HU, E., THOMPSON, J., HORNING, S., TRELA, M., LOWDER, J., LEVY, R. & SKLAR, J. (1985). Detection of B-cell lymphoma in peripheral blood by DNA hybridisation. *Lancet*, **II**, 1093-1095.

JOHNSON, A., CAVALLIN-STÅHL, E. & ÅKERMAN, M. (1991). The significance of B-clonal excess in peripheral blood in patients with non-Hodgkin's lymphoma in remission. *Ann. Oncol.*, **2**, 99-105.

KNOWLES, D., PELICCI, P.-G. & DALLA-FAVERA, R. (1987). Immunoglobulin and T-cell receptor beta chain gene DNA probes in the diagnosis and classification of human lymphoid neoplasia. *Mol. Cell. Probes*, **1**, 15-31.

LINDEMALM, C., MELLSTEDT, H., NILSSON, B., BIBERFELD, P., BJÖRKJOLM, M., CHRISTENSSON, B., HOLM, G. & JOHANSSON, B. (1987). Blood clonal B-cell excess (CBE) at diagnosis in patients with non-Hodgkin's lymphoma (NHL). Relation to clinical stage, histopathology and response to treatment. *Eur. J. Cancer*, **23**, 749-753.

McKENNA, R., BLOOMFIELD, C. & BRUNNING, R. (1975). Nodular lymphoma: bone marrow and blood manifestations. *Cancer*, **36**, 428.

MORRA, E., LAZZARINO, M., ORLANDI, E., INVERARDI, D., CASTELLO, A., COCI, A., MAGRINI, U. & BERNASCONI, C. (1985). Bone marrow and blood involvement by non-Hodgkin's lymphoma: clinicopathologic features and prognostic significance in relationship to the Working Formulation. In *Malignant lymphomas and Hodgkin's Disease: Experimental and Therapeutic Advances*, Cavalli, F., Bonadonna, G. & Rozencweig, M. (eds) pp. 215-224. Martinus Nijhoff: Boston.

PETO, R., PIKE, M., ARMITAGE, P., BRESLOW, N., COX, D., HOWARD, S., MANTEL, N., McPHERSON, K., PETO, J. & SMITH, P. (1977). Design and analysis of randomised clinical trials requiring prolonged observation of each patient. II. Analysis and examples. *Br. J. Cancer*, **35**, 1-39.

PRICE, C., ROHATINER, A., STEWARD, W., DEAKIN, D., BAILEY, N., NORTON, A., BLACKLEDGE, G., CROWTHER, D. & LISTER, T. (1991). Interferon-$\alpha_{2b}$ in the treatment of follicular lymphoma: preliminary results of a trial in progress. *Ann. Oncol.*, **2**, 141-145.

RAVETCH, J., SIEBENLIST, U., KORSMEYER, S., WALDMANN, T. & LEDER, P. (1981). Structure of the human immunoglobulin m locus: characterisation of embryonic and rearranged J and D genes. *Cell*, **27**, 583-591.

THE NON-HODGKIN'S LYMPHOMA PATHOLOGIC CLASSIFICATION PROJECT (1982). National Cancer Institute sponsored study of classification of non-Hodgkin's lymphomas. *Cancer*, **49**, 2112-2135.

WRIGHT, J., POPLACK, D., BAKHSHI, A., REAMAN, G., COLE, D., JENSEN, J. & KORSMEYER, S. (1987). Gene rearrangements as markers of clonal variation and minimal residual disease in acute lymphoblastic leukemia. *J. Clin. Oncol*, **5**, 735-741.